# Unstable SpO_2_ in preterm infants: The key role of reduced ventilation to perfusion ratio

**DOI:** 10.3389/fphys.2023.1112115

**Published:** 2023-02-07

**Authors:** Benjamin Stoecklin, Y. Jane Choi, Theodore Dassios, J. Gareth Jones, Geoffrey G. Lockwood, J. Jane Pillow

**Affiliations:** ^1^ Department of Neonatology, University Children’s Hospital Basel (UKBB), Basel, Switzerland; ^2^ School of Human Sciences, The University of Western Australia, Crawley, WA, Australia; ^3^ Wal-Yan Respiratory Research Centre, Telethon Kids Institute, Nedlands, WA, Australia; ^4^ Neonatal Intensive Care Unit, King’s College Hospital NHS Foundation Trust Denmark Hill, London, United Kingdom; ^5^ Cambridge University Clinical School, Cambridge, United Kingdom; ^6^ Anaesthetic Department, Hammersmith Hospital, London, United Kingdom

**Keywords:** infant, premature, neonatal intensive care unit, pulmonary gas exchange, oxygen inhalation therapy

## Abstract

**Introduction:** Instability of peripheral oxyhemoglobin saturation (SpO_2_) in preterm infants is correlated with late disability and is poorly understood. We hypothesised that a reduced ventilation to perfusion ratio (V_A_/Q) is the key predisposing factor for SpO_2_ instability.

**Methods:** We first used a mathematical model to compare the effects of reduced V_A_/Q or shunt on SaO_2_ stability (SaO_2_ and SpO_2_ are used for model and clinical studies respectively). Stability was inferred from the slope of the SaO_2_ vs. inspired oxygen pressure (*P*_I_O_2_) curve as it intersects the 21 kPa *P*_I_O_2_ line (breathing air). Then, in a tertiary neonatal intensive care unit, paired hourly readings of SpO_2_ and *P*_I_O_2_ were recorded over a 24 h period in week old extremely preterm infants. We noted SpO_2_ variability and used an algorithm to derive V_A_/Q and shunt from the paired SpO_2_ and *P*_I_O_2_ measurements.

**Results:** Our model predicted that when V_A_/Q < 0.4, a 1% change in *P*_I_O_2_ results in >8% fluctuation in SaO_2_ at 21 kPa *P*_I_O_2_. In contrast, when a 20% intrapulmonary shunt was included in the model, a 1% change in *P*_I_O_2_ results in <1% fluctuation in the SaO_2_. Moreover, further reducing the V_A_/Q from 0.4 to 0.3 at 21 kPa *P*_I_O_2_ resulted in a 24% fall in SaO_2_. All 31 preterm infants [mean gestation (±standard deviation) 26.2 (±1) week] had V_A_/Q < 0.74 (normal >0.85) but only two infants had increased shunt at 1.1 (±0.5) weeks’ postnatal age. Median (IQR) SpO_2_ fluctuation was 8 (7)%. The greatest SpO_2_ fluctuations were seen in infants with V_A_/Q < 0.52 (*n* = 10): SpO_2_ fluctuations ranged from 11%–17% at a constant *P*_I_O_2_ when V_A_/Q < 0.52. Two infants had reduced V_A_/Q and increased shunt (21% and 27%) which resolved into low V_A_/Q after 3–6 h.

**Discussion:** Routine monitoring of *P*_I_O_2_ and SpO_2_ can be used to derive a hitherto elusive measure of V_A_/Q. Predisposition to SpO_2_ instability results from reduced V_A_/Q rather than increased intrapulmonary shunt in preterm infants with cardiorespiratory disease. SpO_2_ instability can be prevented by a small increase in *P*_I_O_2_.

## 1 Introduction

Peripheral oxyhemoglobin saturation (SpO_2_) instability may result in more than 100 hypoxemic events per day within the first 8 weeks of life in preterm infants (Di Fiore et al., 2010). Moreover, SpO_2_ instability is poorly documented despite the correlation with an increased rate of late death or disability at 18 months of age (Poets et al., 2015). SpO_2_ instability increases when the infant is in supine compared to the prone position, independent of the mode of respiratory support (Miller-Barmak et al., 2020). A contributing factor for hypoxemic episodes in preterm infants includes sudden decrease in lung volume leading to small airway collapse and intrapulmonary shunt (Bolivar et al., 1995). We have previously shown in adults that SpO_2_ was unstable when ventilation to perfusion ratio (V_A_/Q) was reduced, but was stable with increased shunt (Jones and Jones, 2000).

**FIGURE 1:**
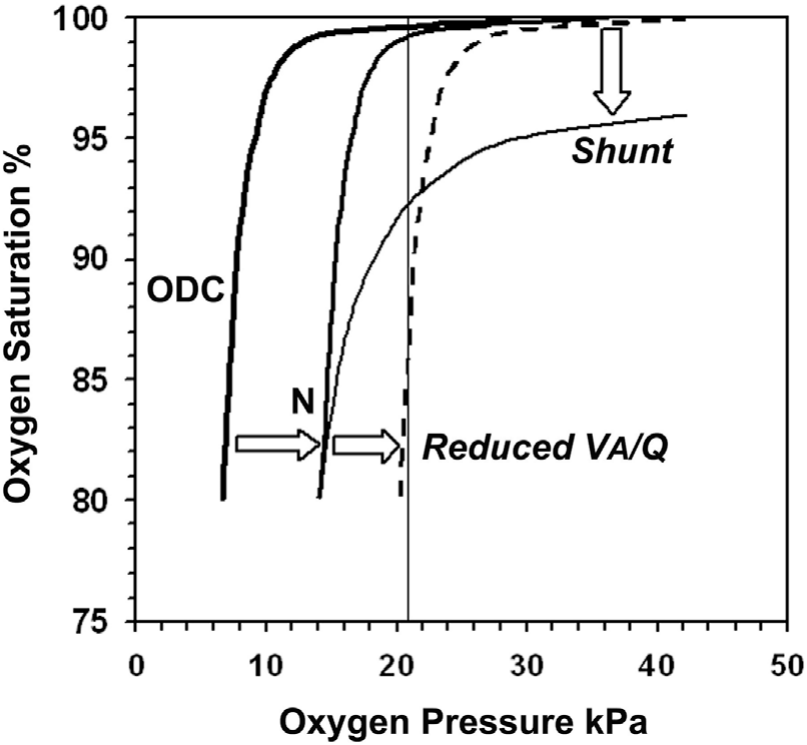
The neonatal oxyhaemoglobin dissociation curve is derived by plotting oxygen saturation (SaO_2_) against arterial oxygen pressure (PaO_2_) and determines the shape of the normal infant SpO_2_ vs. *P*_I_O_2_ curve (N), which is displaced to the right proportional to PCO_2_. Reducing Ventilation to Perfusion ratio (V_A_/Q) shifts the curve further to the right (dashed line). Its slope is now steep as it intersects the 21 kPa *P*_I_O_2_ line where a 1% change in *P*_I_O_2_ results in large SpO_2_ instability. Shunt displaces the plateau downwards with trivial effect on stability.

The concept of V_A_/Q is well established but infrequently used in neonatal practice, as the technics for measuring V_A_/Q in infants are technically difficult e.g. A-a nitrogen difference, or impossible e.g. Multiple Inert Gas Elimination Technique (MIGET) (Corbet et al., 1974; Hand et al., 1990; Roca and Wagner, 1994). Our non-invasive method for measuring V_A_/Q in adults is based on the different effects on SpO_2_ of changing inspired oxygen pressure (*P*_I_O_2_) when either V_A_/Q is reduced or shunt increased (Jones and Jones, 2000). The method depends on the shape of the oxyhaemoglobin dissociation curve (ODC). We adapted this method for preterm infants by using the neonatal rather than the adult ODC as the reference and plotting SpO_2_ at different *P*_I_O_2_ (Lockwood et al., 2014). A computer algorithm derived a model of V_A_/Q and shunt from the paired SpO_2_ and *P*_I_O_2_ dataset. Decreasing V_A_/Q is reflected by a right shift of the SpO_2_ vs. *P*_I_O_2_ curve. The right shift leads to a steeper slope of the curve as it intersects the 21 kPa *P*_I_O_2_ line (*P*_I_O_2_ = F_I_O_2_ x (barometric pressure—saturated water vapour pressure), which means 21 kPa *P*_I_O_2_ is equal to room air at sea level (Figure 1). Any paired SpO_2_ vs. *P*_I_O_2_ measurement located on the steep section of the ODC may predispose the infant to SpO_2_ instability with small changes in alveolar oxygen.

We hypothesised that SpO_2_ instability in preterm infants with cardiorespiratory disease is not an epiphenomenon but an important clinical sign of a reduced V_A_/Q rather than right to left intrapulmonary shunt. We used a new algorithm and routine SpO_2_ monitoring to derive V_A_/Q and shunt.

## 2 Methods

We conducted a prospective observational study in two phases. Firstly, we used a mathematical model of pulmonary gas exchange to examine changes in V_A_/Q or increasing shunt on the slope of the SpO_2_ vs. *P*_I_O_2_ curve.

Secondly, we recorded 24 hourly SpO_2_ vs. *P*_I_O_2_ measurements in extremely preterm infants requiring continuous SpO_2_ monitoring. From these measurements, we derived V_A_/Q and shunt using a pulmonary gas exchange algorithm (Lockwood et al., 2014).

### 2.1 Gas exchange model

We explored the effects of reducing V_A_/Q or increasing shunt on the arterial oxygen saturation (SaO_2_) vs. *P*_I_O_2_ curve using a mathematical model of pulmonary gas exchange described by Olszowka and Wagner (Olszowka et al., 1980). Datasets were generated using the equations implemented on a spreadsheet supplied by Dr AJ Olszowka. The model allowed calculation of exact values of SaO_2_ for a given *P*_I_O_2_. The model lung is subdivided into three compartments: a shunt and two ventilated regions with different alveolar ventilation-perfusion ratios (V_A_/Q). The values of cardiac output, oxygen consumption, hemoglobin concentration, shunt fraction, and the distribution of blood flow and alveolar ventilation to the ventilated compartments can be set. The perfusion of one compartment was set at 90% of non-shunt flow while V_A_/Q was reduced stepwise from 0.85 to 0.3. Shunt was fixed at 2%, *P*_I_O_2_ was varied between 15 kPa and 30 kPa (F_I_O_2_ = 0.15–0.3) and the SaO_2_ was derived at the corresponding *P*_I_O_2_. In the next step, the V_A_/Q was kept constant at 0.85 and the shunt was increased stepwise from 2%–25%. The *P*_I_O_2_ was again varied between 15 and 30 kPa (F_I_O_2_ = 0.15–0.3) and the corresponding SpO_2_ recorded. The slope of the SpO_2_ vs. *P*_I_O_2_ curve was calculated as it intersected the 21 kPa *P*_I_O_2_ line (F_I_O_2_ = 0.21).

### 2.2 Clinical study

#### 2.2.1 Study design

We conducted a prospective observational study at King Edward Memorial Hospital in Perth in Western Australia. The study was approved by the Women and Newborn Health Service Human Research Ethics Committee (HREC:1883EW and 20130193EW) in Perth.

#### 2.2.2 Recruitment period, inclusion and exclusion criteria

Preterm infants born ≤28 weeks’ gestation without major congenital malformations were recruited from the Neonatal Clinical Care Unit at King Edward Memorial Hospital for Women in Perth, Western Australia (KEMH) between 21st August 2017 and the 1st February 2018. All included infants were part of the Preterm Infant Functional and Clinical Outcomes (PIFCO) study (ACTRN12613001062718). We started recruitment for this substudy based on the findings from the main PIFCO cohort, hence the shorter recruitment period and the much smaller number of infants included in the study (Svedenkrans et al., 2019). Informed consent was obtained from parents before the first measurement.

#### 2.2.3 Conduct of study

Infants were assessed at 1 week of age. SpO_2_ measurements were recorded at hourly intervals for 24 h (Masimo Infant Pulse Oximeter Adhesive Sensor RD SET^®^ Inf; Philips Monitor. IntelliVue MP50 or MP70 Neonatal). Measurements were postponed until the following day in infants with changing respiratory support on the day of measurement. F_I_O_2_ was adjusted by the bedside nurses to achieve a SpO_2_ within the target range SpO_2_ 90%–94%. F_I_O_2_ was later converted into *P*_I_O_2_ (*P*_I_O_2_ = F_I_O_2_ x (barometric pressure – saturated water vapour pressure).

#### 2.2.4 Analysis of results

The slope of the SpO_2_ vs. *P*_I_O_2_ curve in preterm infants was analysed using the pulmonary gas exchange algorithm with three lung compartments (Lockwood et al., 2014). Reference normative data of V_A_/Q, right shift of the oxyhemoglobin dissociation curve and right to left shunt in healthy term infants studied in the first week of life were used to quantify the magnitude of the abnormalities in our population of extremely preterm infants (Dassios et al., 2017; Dassios et al., 2019).

#### 2.2.5 Background data

Patient data information including duration of respiratory support and oxygen therapy were collected from the medical charts or the discharge summaries.

#### 2.2.6 Statistical analyses

Study data were collected and managed using Research Electronic Data Capture (REDCap) software hosted at The University of Western Australia. REDCap is a secure, web-based application designed to support data capture for research studies (Harris et al., 2009).

Parametric data are reported as mean and standard deviation (SD) and non-parametric data as median and variance. Statistical analyses included Student’s *t*-test for the comparison of parametric and Mann-Whitney-U test for the comparison of non-parametric data. Data were analysed within SPSS (v25·0·0·1; IBM Corp, United States).

## 3 Results

### 3.1 Gas exchange model

**FIGURE 2:**
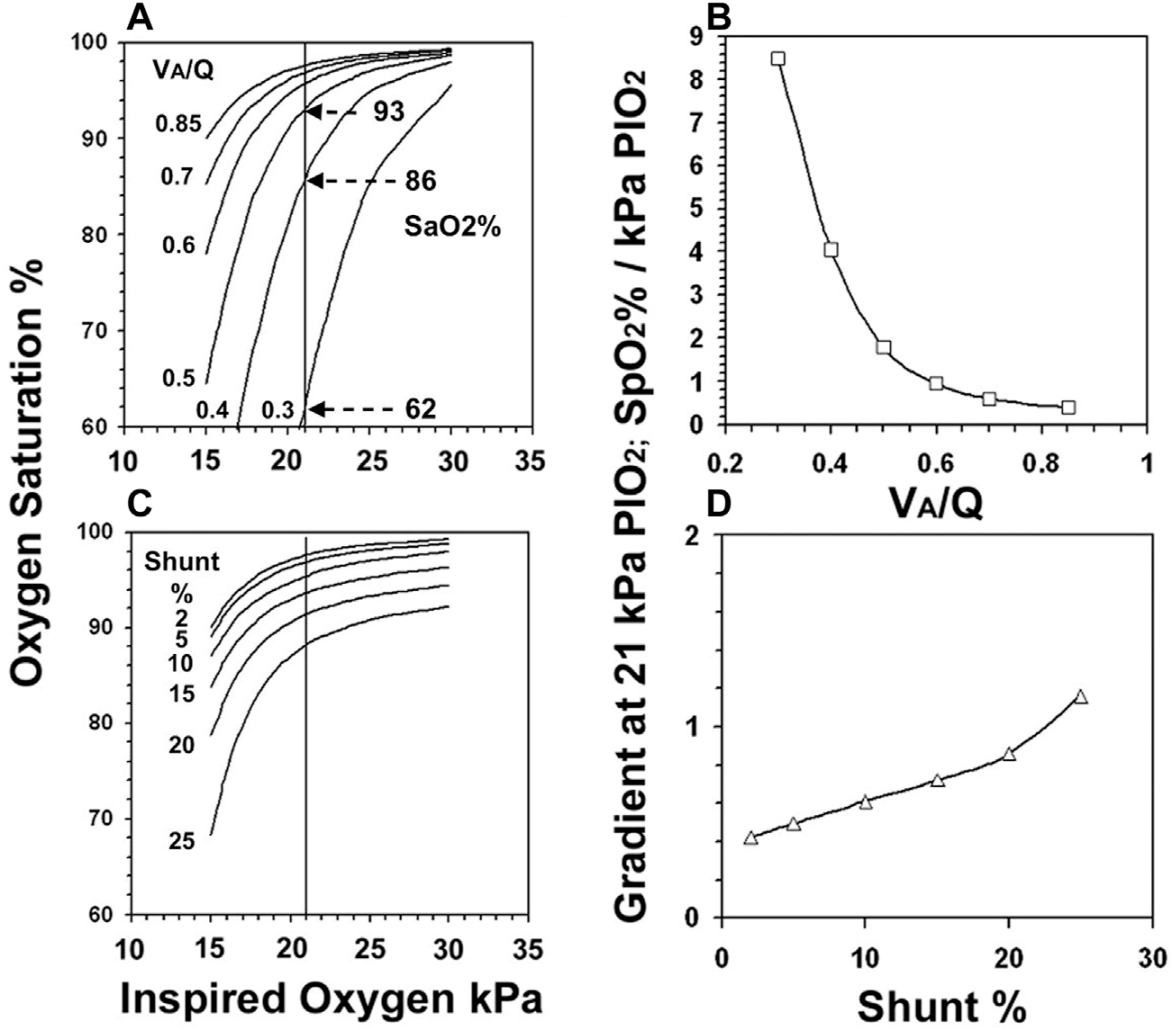
The gas exchange model derived *P*_I_O_2_ vs. SaO_2_ curves when V_A_/Q was reduced from 0.85 to 0.3. (Figure 2A). The slope of the curve as it intercepted the 21 kPa *P*_I_O_2_ line is shown for V_A_/Q (Figure 2B). The *P*_I_O_2_ vs. SaO_2_ curves when shunt increased from 2%–25% (Figure 2C) and their slopes (Figure 2D).

Reducing V_A_/Q shifted the SaO_2_ vs. *P*_I_O_2_ curve to the right so that its slope increased as it intercepted the 21 kPa *P*_I_O_2_ line (Figure 2A). SaO_2_ at the intercept fell in increasingly large increments particularly with V_A_/Q below 0.5: e.g., a 24% SaO_2_ fall when V_A_/Q dropped from 0.4 to 0.3. The effect of V_A_/Q on the slope of the curve (Figure 2B) suggested an unstable SaO_2_ once V_A_/Q fell below 0.5: a 1% change in *P*_I_O_2_ at 0.3 V_A_/Q resulted in a >8% change in SaO_2_.

In contrast, increasing shunt (Figure 2C) displaced the curve downwards but even large changes in *P*_I_O_2_ caused only small changes in both SaO_2_ and slope (Figure 2D): for example in the setting of a 20% intrapulmonary shunt, a 1% change in *P*_I_O_2_ resulted in <1% change in SaO_2_.

### 3.2 Clinical study

We studied 31 extremely preterm infants (mean (SD) 26.2 (1.0) weeks’ gestation) at median (IQR) 1.1 (0.5) weeks’ postnatal age. Eight infants were mechanically ventilated and 23 infants were on non-invasive respiratory support during the measurements. Demographics for this infant cohort are shown in Table 1.

**TABLE 1:** Demographics of infants studied (*n* = 31).

Male (n, %)	20 (64.5)
GA (w)	26.2 ± 1.0
Age at Test (w)	1.1 (0.5)
Weight at birth (g)	890 ± 196
Mechanical Ventilation at test (n, %)	8 (26.0)
Non-invasive respiratory support at test (n, %)	23 (74.0)

GA, gestational age; w, week; g, gram. Values are reported as mean ± SD, median (IQR) or n (%).

**FIGURE 3:**
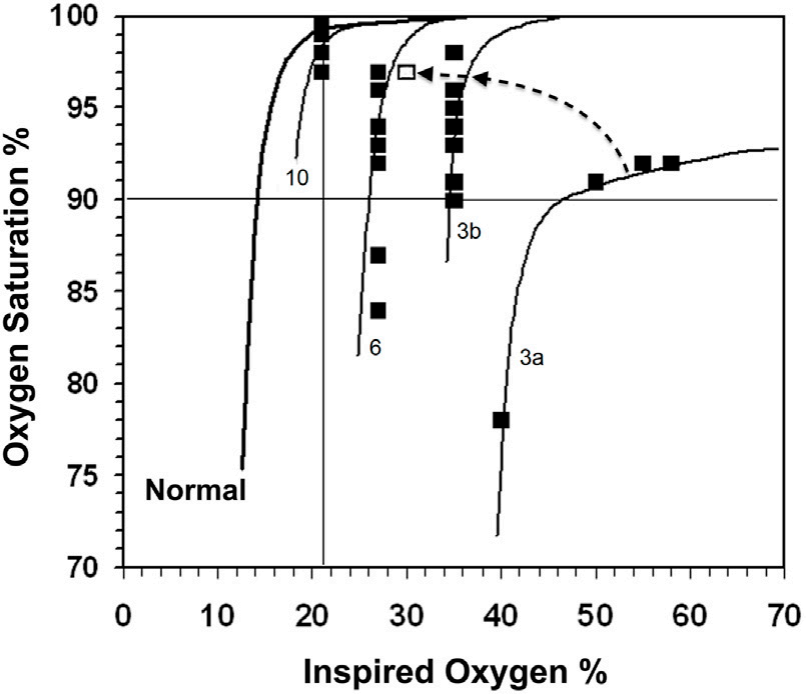
The normal *P*_I_O_2_ vs. SpO_2_ curve is shown on the left. SpO_2_ readings (closed boxes) with the calculated gas exchange model curves for infants 3, 6 and 10. Infant 3 had 27% shunt and V_A_/Q of 0.24 (3a), which resolved during the day to a single V_A_/Q compartment of 0.22 and a SpO_2_ range of 8% (3b) and subsequently into a final point (open box) at the end of the day to almost overlying the V_A_/Q 0.31 line of infant 6 (the changes are indicated by the dashed arrow). Infant 6 initially had a 21% shunt and V_A_/Q of 0.43 (not shown in Figure), which resolved into a V_A_/Q of 0.31 in the 9–24 h study period. Infant 10 had a V_A_/Q of 0.56 and a SpO_2_ range of 3% from 24 SpO_2_ values.

Ten infants received 21 kPa *P*_I_O_2_ throughout the 24 h monitoring period. Median (IQR) *P*_I_O_2_ in the remainder was 28 (Jones and Jones, 2000) % to maintain target SpO_2_ 90%–94%. The number of consecutive hourly readings at the same *P*_I_O_2_ in each infant are shown in Table 2. There were sufficient data to describe the steep part of the SpO_2_ vs. *P*_I_O_2_ curve in all infants in terms of V_A_/Q and shift, but complete characterization of the top end of the curve was constrained by the upper limit of the SpO_2_ target range and therefore restricted *P*_I_O_2_. The V_A_/Q was less than 0.74 in every infant and only two infants (Nr. 3 and 6) had a large shunt resolving after a few hours into predominantly low V_A_/Q compartments. The median (IQR) SpO_2_ of all preterm infants was 8.0 (7.0) % at a constant *P*_I_O_2_ over at least 5 h. At a V_A_/Q < 0.52, in 10 out of 31 infants the variability of the SpO_2_ ranged from 11%–17%, whereas in the four infants with V_A_/Q > 0.52, the range of SpO_2_ recorded was ≤4% (V_A_/Q 0.73, 0.56, 0.57, 0.54). Seven infants had a profound desaturation with a SpO_2_ ≤ 84%. All seven infants had a V_A_/Q ≤ 0.49 and four of them V_A_/Q < 0.31. Examples of the pattern of SpO_2_ in three infants are shown in Figure 3 using the gas exchange algorithm (Lockwood et al., 2014) and a format similar to Figure 1.

**TABLE 2:** Measurements of included infants.

Infant number	MV	V_A_/Q	Shift kPa	*P*_I_O_2_kPa	Number of SpO_2_ at that *P*_I_O_2_	Min SpO_2_%	Med SpO_2_%	Range SpO_2_%	Variance SpO_2_%
1	N	0.52	11.6	21	24	93	98	14	6.8
2	N	0.49	12.4	22	15	83	94	15	11.0
3	Y	0.22	27.5	35	14	90	94	8	6.0
4	N	0.21	28.8	38	5	88	95	9	28.8
5	N	0.27	22.8	30	14	87	93	9	5.9
6	Y	0.31	19.2	27	13	84	96	13	16.9
7	N	0.5	12.0	21	24	93	97	6	3.3
8	N	0.73	8.3	21	24	96	98	4	1.5
9	Y	0.46	13.3	21	19	91	95	6	3.3
10	N	0.56	10.8	21	24	97	98	3	0.6
11	N	0.57	10.6	21	24	96	99	4	1.7
12	N	0.54	11.3	21	24	96	98	4	1.6
13	Y	0.35	17.5	25	15	91	94	5	2.1
14	N	0.3	20.0	28	17	90	96	8	4.0
15	N	0.39	15.4	24	8	84	91	14	22.3
16	N	0.49	12.3	21	24	93	96	7	2.8
17	N	0.3	20.2	28	16	91	95	5	2.7
18	N	0.49	12.4	21	19	92	96	7	4.1
19	Y	0.24	25.0	32	10	84	93	14	17.5
20	Y	0.27	22.6	30	12	88	94	8	7.7
21	N	0.28	22.7	30	12	88	93.5	9	8.4
22	Y	0.3	20.1	30	9	89	95	8	6.3
23	N	0.44	13.8	21	14	81	93	16	16.8
24	N	0.27	22.8	30	10	81	94	17	22
25	N	0.47	12.8	21	24	91	95	8	3.7
26	N	0.52	11.6	21	24	95	97.5	4	11.6
27	N	0.35	17.3	25	13	92	94	6	17.3
28	N	0.39	15.5	23	10	86	94	12	15.5
29	N	0.47	12.8	21	24	92	96	5	12.6
30	N	0.27	22.8	30	13	84	93	12	22.8
31	Y	0.29	20.8	28	9	87	94	11	20.8

Spot reading of SpO_2_ and *P*_I_O_2_ at 1 h intervals within a 24 h monitoring period. Infant 3 had four readings at *P*_I_O_2_ 78, 92, 92 & 91 kPa from 3–6 h to derive a 27% shunt, V_A_/Q 0.24 and shift of 34.1 kPa. From 10–24 h the 27% shunt resolved at constant 35 kPa *P*_I_O_2_ to a single compartment V_A_/Q of 0.22 and 27.4 kPa shift. MV, mechanical ventilation.

One infant (Nr. 3) required a *P*_I_O_2_ > 50 kPa to maintain SpO_2_ within the target range. This infant had an initial large shunt and greatly reduced V_A_/Q, which later resolved into a single low V_A_/Q compartment. Infant Nr. 3, 6 and 10 are described in detail in the legend to Figure 3.

**FIGURE 4:**
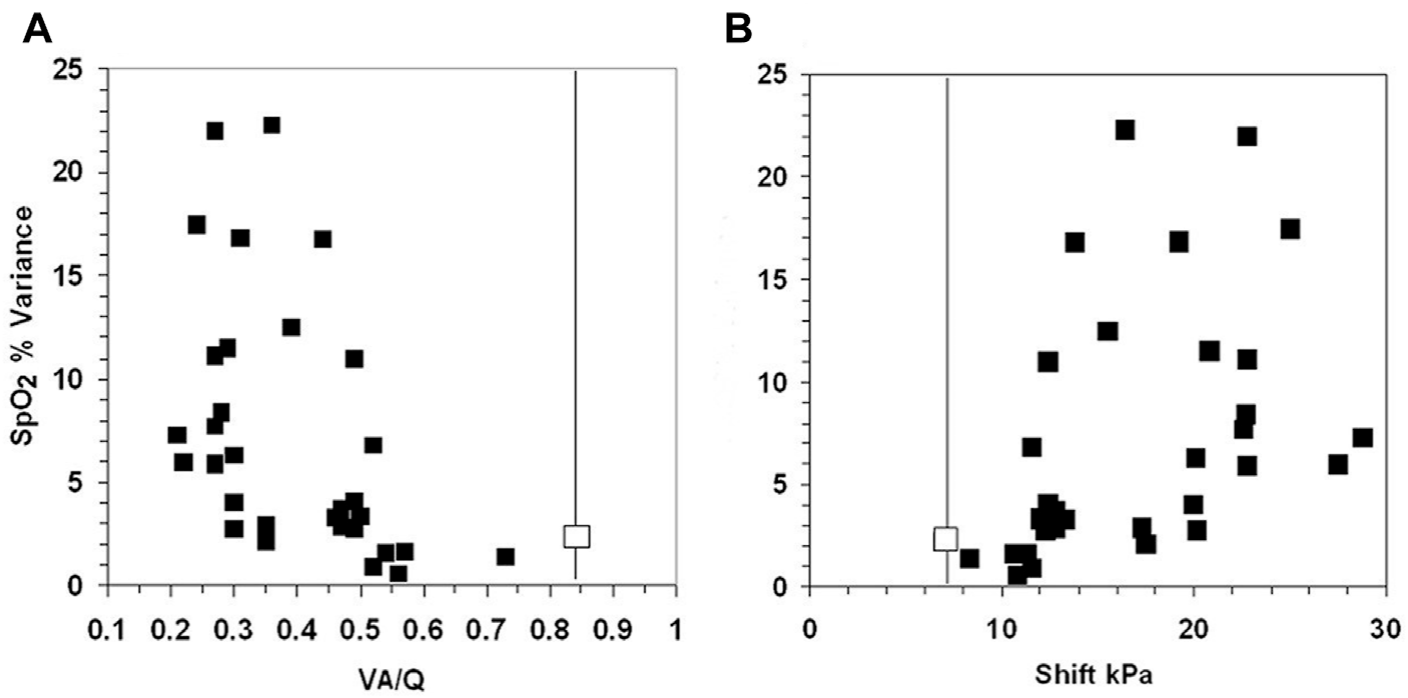
The fall in V_A_/Q below 0.52 (Figure 4A) and an increase in shift >11 kPa (Figure 4B), were associated with an abrupt widening of the variance of SpO_2_. The two uppermost points have a range of 16% and 17% SpO_2_ in infants 23 and 24. The normal values for V_A_/Q and shift are shown by the vertical line. The normal variance for SpO_2_ is 2.2 shown by the contrasting boxes on the vertical line (Dassios et al., 2017; Dassios et al., 2019).

The 10 infants exhibiting the most variable SpO_2_ (range >10% in SpO_2_) were equally distributed between those breathing spontaneously (*n* = 5) and those requiring mechanical ventilation (*n* = 5) (Table 1). The increased variance in SpO_2_ as V_A_/Q falls below 0.5 is illustrated in Figure 4A shift increases >11 kPa in Figure 4B.

## 4 Discussion

We suggest that a reduced V_A_/Q predisposes preterm infants to SpO_2_ instability. The pulmonary gas exchange model showed that reducing V_A_/Q shifted the *P*_I_O_2_ vs. SaO_2_ curve to the right, whereas shunt displaced the curve downwards. Right shift increased the effective curve slope up to 16 times where it intercepted the 21 kPa *P*_I_O_2_ line e.g., breathing room air. The right shift predicted large fluctuations in SaO_2_ with small changes in alveolar oxygen. Reducing V_A_/Q from 0.4 to 0.3 predicted a 24% fall in SaO_2_ breathing air. In contrast a large shunt had little effect on curve slope and a small fall in SaO_2_.

These predictions were confirmed in extremely preterm infants by routine monitoring of SpO_2_ and *P*_I_O_2_. The paired SpO_2_ vs. *P*_I_O_2_ measurements were used to derive V_A_/Q, right shift and shunt using Lockwood’s algorithm (Lockwood et al., 2014). All infants had a reduced V_A_/Q. The SpO_2_ was unstable with a median (IQR) 8.0 (7.0) % at a constant *P*_I_O_2_ compared to a mean (±SD) of 3% (±1.5%) in healthy newborn infants breathing room air (Dassios et al., 2017; Dassios et al., 2019). Infants with V_A_/Q < 0.52 had considerable increase in SpO_2_ variability, seven infants had episodes with SpO_2_ ≤ 84%, whilst four of these infants were dependent on supplemental oxygen ≥27%. Two infants had transient shunt lasting for a few hours, which was not associated with SpO_2_ instability. In the remaining infants, the *P*_I_O_2_ was insufficiently high to fully characterize shunt due to the SpO_2_ target range (90%–94%).

The non-invasive method for deriving V_A_/Q and shunt normally depends on varying *P*_I_O_2_ (Rowe et al., 2010; Bamat et al., 2015; Dassios et al., 2017; Dassios et al., 2019; Svedenkrans et al., 2019; Bamat et al., 2022). More recently, we showed that V_A_/Q and/or shift can be derived from serial SpO_2_ measurements at a fixed *P*_I_O_2_ (Svedenkrans et al., 2019; Stoecklin et al., 2021). A reduced V_A_/Q lowers SpO_2_ at any given *P*_I_O_2_ and increases the effective slope of the SpO_2_ vs. *P*_I_O_2_ curve, but does not by itself make SpO_2_ unstable unless V_A_/Q itself is unstable (Jones, 2021). The reduced V_A_/Q amplifies the destabilizing effects on SpO_2_ during changes in cardiac output, ventilation, central or obstructive apnea and posture. Interestingly, the 10 infants with the most unstable SpO_2_ were equally distributed between those breathing spontaneously and those on mechanical ventilation. Reduced V_A_/Q can lead to SpO_2_ instability independent of the mode of respiratory support used in an infant.

In agreement with our results, a reduced V_A_/Q in adults leads to SpO_2_ instability breathing room air. By adding a time dimension, the different effects of V_A_/Q or shunt in individual adult patients breathing air is more clearly illustrated by dynamic waterfall plots of continuously monitored SpO_2_ (Entwistle et al., 1991; Jones and Jones, 2000; Jones, 2021) than do “static” histograms (Borenstein-Levin et al., 2020). A typical example of unstable SpO2 in a patient with reduced V_A_/Q breathing air is shown in Figure 3 in Ref (Jones, 2021). This patient had blunt curves with SpO_2_ fluctuating from 77% to 95%. In contrast, a patient with increased shunt had superimposed stable SpO_2_ peaks within a much narrower SpO_2_ range.

We note that the right to left shunt in our study might include some element of cardiac shunting occurring *via* an open ductus arteriosus. Our method does not allow differentiation between intrapulmonary or cardiac shunt. However, in the majority of preterm infants the ductus arteriosus would have functionally closed on day seven of life. In the few infants with a persistent ductus arteriosus, at day seven of life a predominantly left to right shunt would be expected which would not affect our calculations (de Klerk et al., 2020).

The clinical applicability of our findings is that routine measurements of SpO_2_ and *P*_I_O_2_ can be analysed with our algorithm to derive V_A_/Q and shunt, thereby characterizing respiratory disease in preterm infants. V_A_/Q and shunt incorporate information on the mechanisms of hypoxemia and explain SpO_2_ instability. Consequently, SpO2 instability can be prevented by a small increase in *P*_I_O_2_.

## 5 Conclusion

In conclusion, we reported that predisposition to oxygen saturation instability in preterm infants results from a reduced ventilation to perfusion ratio rather than from increased intrapulmonary shunt. We have highlighted how routine monitoring can be used to derive non-invasive measurements of oxygenation impairment in preterm infants. Future research should aim at strategies to not only detect SpO_2_ instability, but to also prevent such instability.

## Data Availability

The raw data supporting the conclusion of this article will be made available by the authors, without undue reservation.
